# Inflammatory Cytokine and Chemokine Patterns in Paediatric Patients with Suspected Serious Bacterial Infection

**DOI:** 10.3390/medicina55010004

**Published:** 2019-01-03

**Authors:** Linda Rautiainen, Jana Pavare, Ilze Grope, Peteris Tretjakovs, Dace Gardovska

**Affiliations:** 1 Lapland Central Hospital, Department of Paediatrics, Rovaniemi 96101, Finland; 2 Department of Paediatrics, Riga Stradins University, Riga LV-1007, Latvia; jana.pavare@rsu.lv (J.P); ilze.grope@rsu.lv (I.G.); dace.gardovska@rsu.lv (D.G.); 3 Department of Biochemistry and Physiology, Riga Stradins University, Riga LV-1007, Latvia; peteris.tretjakovs@rsu.lv

**Keywords:** serious bacterial infection, children, inflammation, cytokine

## Abstract

*Background and objectives:* In children, acute infection is the most common cause of visits to the emergency department. Although most of them are self-limiting, mortality due to severe bacterial infections (SBI) in developed countries is still high. When the risk of serious bacterial infection is too high to ignore, yet too low to justify admission and hospital observation, clinicians try to improve diagnostic accuracy by performing various laboratory tests. The aim of the study was to investigate whether an early inflammatory cytokine and chemokine panel can add information in diagnostics of SBI and assessment of efficacy of early therapies in hospitalized children with fever. *Methods:* This study included 51 children with febrile infections that were admitted to the emergency department (ED). Clinical examination and microbiological and radiological tests were used as reference standards for the definition of SBI. Study population was categorized into two groups: (1) patients with SBI (*n* = 21); (2) patients without SBI (*n* = 30). Inflammatory cytokine and chemokine panels were analyzed from the first routine blood samples at hospital admission and after 24 h. *Results:* Out of 12 cytokines and chemokines, only Eotaxin and granulocyte colony-stimulating factor (G-CSF) had statistically significant differences between groups at the time of inclusion. Receiver operator characteristic analysis to predict SBI showed an area under the curve (AUC) of 0.679 for G-CSF. *Conclusions:* Analysis of inflammatory cytokine profiles may provide additional information in early diagnostics of SBI.

## 1. Introduction

In children, acute infection is the most common cause of visit in the primary care or emergency department, and although most are self-limiting, mortality due to severe bacterial infections (SBI) in developed countries is still high [1]. Due to low incidence, non-specific initial presentation, and risk of rapid deterioration, the assessment of children with acute infection is difficult [2]. There have been studies and reviews about clinical decision-making rule and laboratory tests which could help distinguish between self-limiting, viral infection, and SBI [2,3].

In 2013, Verbakel et al. published a systematic review about accuracy of clinical prediction tools for SBI, where they conclude that none of the clinical predictions’ tools examined provided perfect clinical accuracy [2]. Van den Bruel et al. have published a systematic review about diagnostic value of laboratory tests in identifying SBI; their conclusion was that measurement of inflammatory markers in emergency department can be diagnostically useful, but different cut-off values should be used whether clinicians want to rule out or confirm SBI [3].

In 2002, The International Consensus Conference on Pediatric Sepsis and Organ Dysfunction produced consensus clinical definitions of systemic inflammatory response syndrome (SIRS) and sepsis in children [4], which became a landmark of diagnostics of paediatric sepsis, and was thought to aid in identifying serious bacterial infections. According to this, patients were not required to have positive blood cultures to be diagnosed with sepsis. However, it has its limitations, which include an excessive focus on inflammation cascade and inadequate specificity and sensitivity of SIRS, as well as a model of sepsis following a continuum through severe sepsis to septic shock [5].

Even after thorough clinical assessment, clinicians are left with uncertainty where the risk of serious bacterial infection is too high to ignore, yet too low to justify admission and hospital observation. In these cases, clinicians try to improve diagnostic accuracy by performing various laboratory tests [3].

C reactive protein (CRP) is the most widely used diagnostic and prognostic marker, despite its limitation due to late appearance and persistence for relatively longer periods [6]. In addition, CRP and procalcitonin (PCT) have limited ability to distinguish SBI from other nonbacterial inflammatory conditions, and they are not useful in predicting outcome [7]. Use of “emerging” biomarkers for early diagnosis of SBIs and sepsis has been widely studied. Meem et al., in a review of published data about diagnostic markers of neonatal sepsis, have found PCT, interleukin 6 (IL-6), interleukin 8 (IL-8), interferon gamma (IFN-γ), tumor necrosis factor alpha (TNF-α), cluster of differentiation 64 (CD64), and soluble intercellular adhesion molecules (sICAM) to be the most promising [6]. IL-6 and IL-8 have been shown to have elevated levels in sepsis, and are also associated with severity and outcome [8].

Furthermore, there are published data confirming that combining information from several inflammatory markers improves accuracy in distinguishing bacterial and nonbacterial causes of inflammation [9]. A good biomarker for diagnostics of SBI would have diagnostic, prognostic, and follow-up of therapy characteristics, and would also be rapidly and easily used in clinical practice [10].

In light of this, the aim of the study was to investigate whether an early inflammatory cytokine and chemokine panel could provide additional information in diagnostics of SBI, and also help to assess the efficacy of therapies in children with SBI.

## 2. Materials and Methods

Patient recruitment took place from October 2011 to December 2013 in Children’s Clinical University hospital, Riga, Latvia.

The inclusion criteria were: hospitalized children with fever, aged from 1 month to 17 years. Patients were assessed by physicians according to hospital’s standard of care.

SBI, at the emergency department and during the later revision of clinicians, was defined based on available clinical, imaging, and later also on microbiological data, as having either bacteraemia, pneumonia (radiographically confirmed), meningitis, osteomyelitis, complicated urinary tract infection, skin/soft tissue infection, culture positivity of usually sterile body fluid, or clinical diagnosis by radiology (pneumonia, osteomyelitis, intra-abdominal infection) [11]. The group of patients with fever but without SBI included children with upper respiratory tract infections and viral gastroenteritis.

Patients with SBI were divided into age groups (1–12 months, 12–60 months, 60–144 months and 144–216 months) and patient controls without SBI of the same age group were matched.

The exclusion criteria were antibacterial therapy within the last 48 h, immunodeficiency, chronic liver or kidney illness, vaccination within 5 days before the start of the illness, congenital metabolic defects, chromosomal anomalies, and use of corticosteroids or immunosuppressant medications. Other exclusion factors from the study were obesity, diabetes mellitus, chronic inflammatory diseases, such as rheumatoid arthritis, systemic lupus erythematosus, vasculitis, inflammatory bowel disease, heart diseases, renal or liver diseases, or malignancies and other diseases which are known to be associated with significant changes of anti- and pro-inflammatory biomarkers, including surgery or trauma within the preceding 30 days.

Informed consent was obtained from patients’ parents and additionally from patients themselves, if applicable. The study protocol was approved by the Committee of Ethics of Riga Stradin’s University (No 2./06.10.2011). All patients had received the standard of care according to hospital guidelines.

All patients had blood samples drawn at the time of inclusion and after 24 h. Plasma CRP were measured in all patients according to hospital’s standards. Patients also had 5 mL of blood drawn for serum centrifugation, after which the serum was then frozen, and all patients’ samples were analysed at once. In all patients, there were the same inflammatory cytokine and chemokine panels (soluble apoptosis- stimulating fragment (sFAS), soluble vascular cell adhesion molecule (sVCAM-1), total plasminogen activator inhibitor type 1 (tPAI1), IL-8, INF gamma, tumor necrosis factor alpha (TNF-α), Eotaxin-1, granulocyte colony-stimulating factor (G-CSF), interleukin-1 receptor antagonist (IL-1ra), interferon-inducible protein-10 (IP-10), monocyte chemoattractant protein-1 (MCP-1). Cytokine panels were analyzed using Luminex^®^ xMAP^®^ technology, which is a multiplex assay approach (Luminex 200™, Merck Millipore, Germany).

Statistical analysis was performed using IBM SPSS 22, and included descriptive statistics with median values, standard deviation, and range for continuous variables where appropriate. To test for differences between the compared groups, Mann-Whitney tests were used for continuous variables as appropriate, and the chi-squared test was used for categorical variables. Receiver-operating characteristic (ROC) curves were developed for each of the continuous biomarkers presenting the area under the curve, including the 95% CI for each biomarker. The Youden’s index was used to determine the cut-off values for each indicator to determine sensitivity and specificity. A two-tailed *p* value <0.05 was statistically significant.

## 3. Results

In total, out of 140 patients with fever who were hospitalized from the emergency department, 124 met the inclusion criteria. 54 patients, whose parents consented to the study, were included. Patients were then divided into 2 groups based on possible presence or absence of SBI after clinical evaluation. At the end of the study data from 3 patients was discarded, as their inclusion samples were haemolytic, thus, not suitable for analysis.

The baseline characteristics of the study sample are depicted in Table 1.

In the SBI group, 15 patients had pneumonia, 3 had complicated urinary tract infections, 2 patients had osteomyelitis, and one had meningitis. In the SBI group blood cultures were drawn in 38.1% (8), only one patient with osteomyelitis had a positive blood culture for *Staphylococcus aureus*. There were statistically significant differences between SBI and non-SBI groups in the length of hospitalization and antibacterial therapy.

All patients were discharged from the hospital at the end of the treatment.

At the inclusion of the study for all patients, all inflammatory markers were tested, and groups were compared. The numerical values of inflammatory cytokines in both groups can be seen in Table 2.

**Figure 1:**
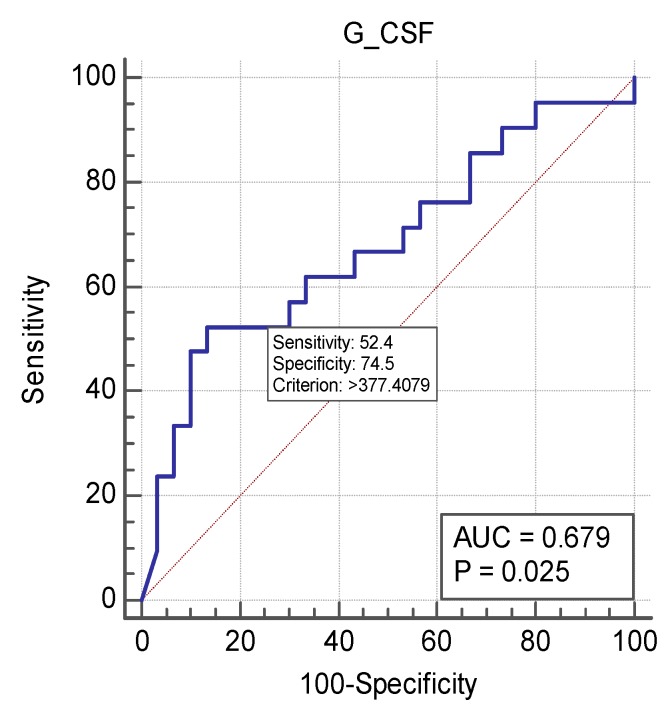
Area under the receiver-operating characteristic (ROC) curve for granulocyte colony-stimulating factor (G-CSF).

We found statistically significant differences in Eotaxin and G-CSF levels between groups at the time of inclusion. Eotaxin levels were in fact higher in patients without SBI. As G-CSF levels were significantly higher in patients with SBI, we calculated its ROC-curve, which is graphically depicted in Figure 1.

G-CSF area under the ROC curve (AUC) was 0.679 (±0.08, 95% CI 0.534–0.803) with a *p* = 0,025, it had sensitivity of 52.38% and specificity of 86.67%.

As the next step, we performed paired sample analysis using Wilcoxon signed rank test and compared the significance of changes of these markers at the time of inclusion and after 24 h of treatment. There were no significant changes in inflammatory cytokines in a 24 h time period in the non-SBI group. In the SBI group, statistically significant changes between time of inclusion and 24 h the following markers showed sFAS (*p* = 0.041), IL-10 (*p* = 0.027), INFγ (*p* = 0.016), Eotaxin (*p* = 0.027), G-CSF (*p* < 0.001), and IP-10 (*p* = 0.012). Eotaxin showed significant increase in a 24 h time period, whilst all the other cytokines decreased after 24 h. The numerical values are depicted in Table 3.

## 4. Discussion

The diagnostics of SBI are still challenging although there are new tests and clinical scores available. The diagnosis of SBI is complicated by its non-specific clinical symptoms, and their variability; failure to diagnose and promptly treat SBI results in significant morbidity and mortality [12]. In addition, the complex pathophysiology and great patient-to-patient variability in SBIs needs to be considered.

When comparing inflammatory cytokine profiles, we found statistically significant differences between groups only in levels of Eotaxin and G-CSF. There were also significant differences in some of the inflammatory cytokines after 24 h of inclusion, and, of course, therapy.

Eotaxin-1 has not been studied either as a diagnostic nor as a prognostic marker for SBIs. There are publications about levels of Eotaxin-1 in parasitic infections [13], in which patients with tuberculosis had significantly higher levels than controls[14]. There have been no large studies about its use on diagnostics of SBI. Eotaxin-1 has also been studied in cases of serious acute respiratory illness, where its levels were significantly higher if a respiratory virus was present in the broncho-alveolar lavage fluid [15]. This could partially explain our results, where Eotaxin-1 was significantly higher in patients without SBI, as this group was mainly composed of patients with viral respiratory infections. It is unclear why there was a significant increase in Eotaxin levels in children with SBI after 24 h of therapy, whilst all the other significant changes between cytokine levels showed a decrease.

In patients with SBI, several inflammatory markers (sFAS, Eotaxin) showed a significant increase after 24 h. Although all patients in this group received antibacterial therapy, it is unclear how this inflammatory response correlates with therapy. G-CSF, IL-10, INF γ and IP-10 showed a significant decrease over 24 h in patients with SBI.

G-CSF has been largely studied as a treatment for sepsis and septic shock. In preterm neonatal patients, rhG-CSG adjunctive therapy has been shown to decrease mortality [16]. In an animal study, performed by Gao et al., a combination of biomarkers was measured in rats after induced sepsis. The results showed that a combination of different biomarkers improved the diagnostic accuracy [17]. Increased G-CSF concentrations have been shown to be predictive of worsening organ dysfunction in sepsis and had good accuracy in predicting early mortality [18]. G-CSF has not been studied as a diagnostic marker.

There are published data about IP-10 as a possible diagnostic marker. It has been studied as a diagnostic marker in urine in cases of neonatal sepsis, where it showed statistically significant increase in neonates with bacterial infections [19].

IL-10, an anti-inflammatory cytokine which prevents the excess pro-inflammatory response, has been widely studied in different populations. High levels of IL-10 in patients with septic shock have been correlated with poor prognosis. However, an appropriate IL-10 response has been shown to have a protective effect against inflammation. Our patients with SBI had a significant decrease in IL-10 levels after 24 h of therapy. As we had no mortality in this patient group, we can speculate that this decrease is a logical step in the inflammatory pathway as a normal response to therapy.

This study had several limiting factors, the most important being the small patient population. This is mostly attributable to difficulties of patient enrolment and technical difficulties of venous blood sampling in children. In addition, we had no control group of healthy children, which could provide baseline levels for inflammatory cytokines. For more accurate determination of significance of these markers in diagnostics of SBI, further studies with bigger patient population are necessary.

## 5. Conclusions

This study added information about inflammatory cytokine panels in hospitalized children with and without SBI and their dynamic changes after 24 h of therapy. We also detected significant changes in levels of G-CSF between patients with and without SBI, which could improve diagnostic accuracy in patients with SBI.

## Figures and Tables

**Table 1:** Characteristics of study population.

Title 1	Severe Bacterial Infection (SBI) Patients (*n* = 21)	Patients without SBI (*n* = 30)
Age, median, months (±SD)	43.0 ± 63.9	30.5 ± 59.62
Sex, percent (*n*)	42.9% (9) males 57.1% (12) females	63.3% (19) males 36.7% (11) females
Inclusion day after symptom onset, median (±SD)	5 ± 2.48	3 ± 3.50
C-reactive protein (CRP), median (±SD), mg/L	131.95 ± 111.94	35.65 ± 61.3
Length of hospitalization, median (±SD)	8 ± 7.81	3 ± 2.33
Antibacterial therapy	100% (21)	50% (15)

**Table 2:** Median, range and comparison of cytokines and chemokines between patients with and without severe bacterial infections (SBI) at the time of inclusion.

Inflammatory Cytokines, Median (min–max) pg/mL	SBI (*n* = 21)	Patients without SBI (*n* = 30)	*p* Value
Soluble apoptosis-stimulating fragment (sFas)	3356.23 (1606.69–6791.45)	3740.37 (1925.82–6832.95)	*p* = 0.153
Soluble vascular cell adhesion molecule (sVCAM1)	1306.07 (568.63–5042.88)	1010.95 (392.91–4197.20)	*p* = 0.243
Total plasminogen activator inhibitor type 1 (tPAI-1)	147.80 (72.01–353.81)	136.43 (56.80–327.74)	*p* = 0.389
Interleukin 8 (IL-8)	12.6 (1.56–158.57)	10.2 (4.00–35.70)	*p* = 0.723
Interleukin 10 (IL-10)	30.10 (16.22–7127.79)	40.35 (9.71–3365.79)	*p* = 0.841
Interferon gamma (INF-gamma)	16.9 (0.13–172.36)	13.6 (0.34–838.20)	*p* = 0.688
Tumor necrosis factor alpha (TNF-alfa)	13.97 (0.67–100.41)	13.99 (6.42–35.24)	*p* = 0.566
Eotaxin	50.23 (14.80–107.76)	73.61 (13.50–107.76)	*p* = 0.035
Granulocyte colony-stimulating factor (G-CSF)	504.69 (18.88–10,000)	187.27 (29.19–10,000)	*p* = 0.031
Interleukin-1 receptor antagonist (IL1ra)	4.94 (3.20–158.60)	16.1 (2.00–125.17)	*p* = 0.601
Interferon-inducible protein-10 (IP10)	977.78 (218.74–10,000)	1070.93 (100.60–10,000)	*p* = 0.836
Monocyte chemoattractant protein-1 (MCP1)	319.05 (126.35–4788.12)	411.28 (39.15–5763.23)	*p* = 0.168

**Table 3:** Comparison of inflammatory cytokine patterns at the time of inclusion and after 24 h in patients with severe bacterial infections (SBI).

Inflammatory Cytokines, Median (min-max) pg/mL	Time of Inclusion	After 24 h	*p* Value
Soluble apoptosis-stimulating fragment (sFas)	3356.23 (1606.69–6791.45)	3530.31 (1925.82–7553.48)	*p* = 0.041
Soluble vascular cell adhesion molecule (sVCAM1)	1306.07 (568.63–5042.88)	919.10 (628.18–4859.74)	*p* = 0.078
Total plasminogen activator inhibitor type 1 (tPAI-1)	147.80 (72.01–353.81)	154.99 (92.44–286.00)	*p* = 0.383
Interleukin 8 (IL-8)	12.6 (1.56–158.57)	8.78 (2.89–105.36)	*p* = 0.0383
Interleukin 10 (IL-10)	30.10 (16.22–7127.79)	26.12 (6.47–3385.94)	*p* = 0.027
Interferon gamma (INF-gamma)	16.9 (0.13–172.36)	5.18 (0.13–120.94)	*p* = 0.016
Tumor necrosis factor alpha (TNF-alfa)	13.97 (0.67–100.41)	12.42 (4.74–73.12)	*p* = 1.000
Eotaxin	50.23 (14.80–107.76)	64.02 (20.49–122.77)	*p* = 0.027
Granulocyte colony-stimulating factor (G-CSF)	504.69 (18.88–10,000)	129.75 (29.19–2074.56)	*p* < 0.001
Interleukin-1 receptor antagonist (IL1ra)	4.94 (3.20–158.60)	3.20 (1.66–77.85)	*p* = 0.146
Interferon-inducible protein-10 (IP10)	977.78 (218.74–10,000)	576.579 (161.67–10,000)	*p* = 0.012
Monocyte chemoattractant protein-1 (MCP1)	319.05 (126.35–4788.12)	309.16 (65.71–4327.81)	*p* = 0.383
